# Supplementary material for: Reverse transcriptase inhibitors enable the generation of fertile spermatids from fetal mouse testes in vitro
Source: Commun Biol. 2026 Jan 27;9:329. doi: 10.1038/s42003-026-09613-y (PMC12953628; doi:10.1038/s42003-026-09613-y)
Supplement: Supplementary file 2 — Supplementary Information [file 42003_2026_9613_MOESM2_ESM.pdf]

Supplementary Fig. 1

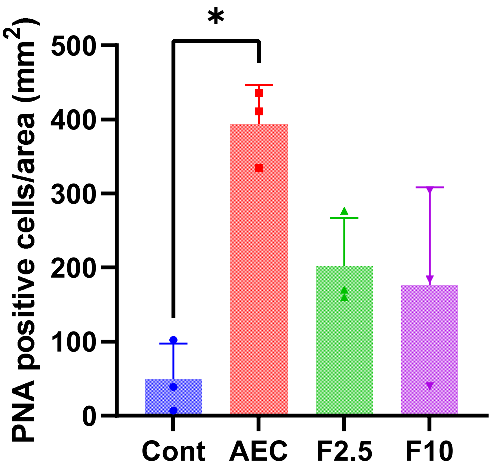

Supplementary Fig.1: Number of PNA-positive cells per testicular tissue area under various culture conditions.
The cell count was normalized to the area of the testicular tissue. These results were similar to those from the analysis of PNA-positive cells per seminiferous tubule (Fig. 3f). Data are presented as mean ± s.d. *$p < 0.05$ (Dunn's multiple comparison test).

Supplementary Fig. 2

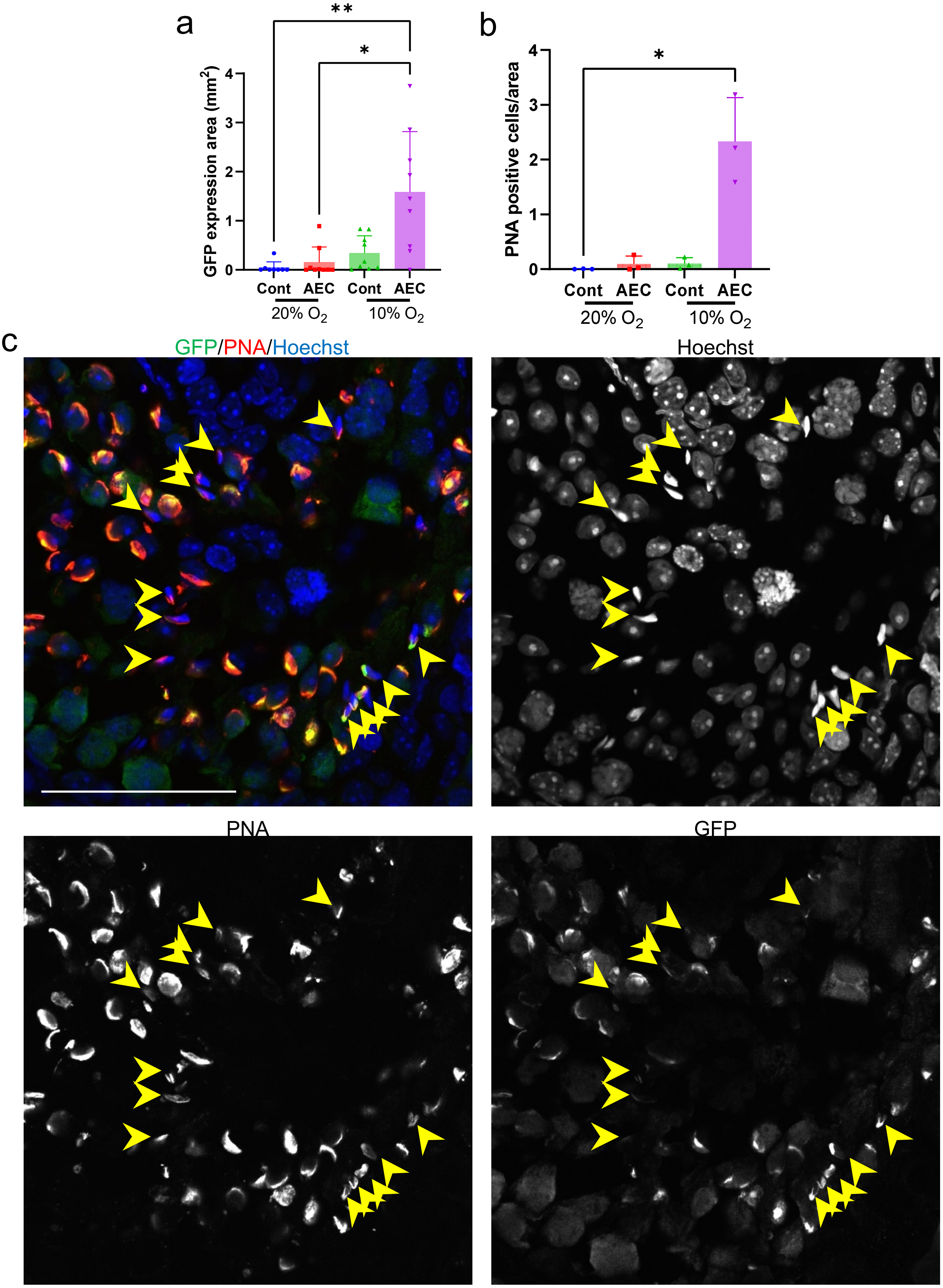

Supplementary Fig. 2: Induction of spermatogenesis under hypoxic conditions.

a Area of GFP expression under each culture condition. The graph shows the data from Fig. 4b plotted as the absolute GFP-positive area.

b Number of PNA-positive cells per testicular tissue area under various culture conditions. Data are presented as mean ± s.d. *$p < 0.05$ (Dunn's multiple comparison test).

c Elongating spermatids develop in the 10% O_2, AEC medium condition. High-magnification images of the region indicated by the yellow dotted line in panel d are shown. Individual channels (Hoechst, PNA, GFP) are displayed in grayscale. An arrowhead points to a cell exhibiting a highly condensed nucleus and a narrow, elongated PNA signal, features that are characteristic of an elongating spermatid. Scale bar = 50 μm.

Supplementary Fig. 3

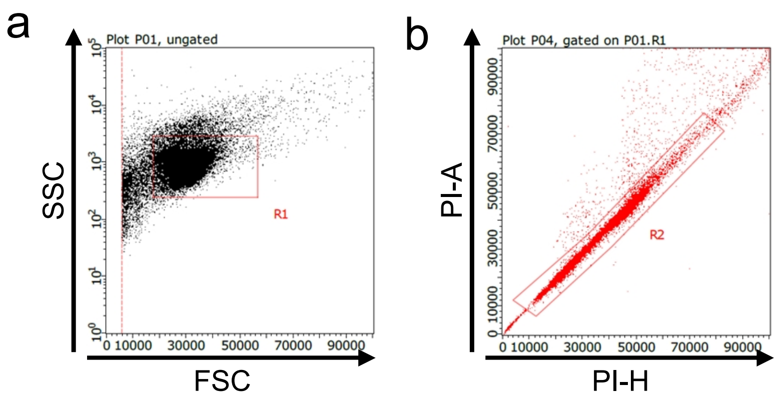

Supplementary Figure 3: Gating strategy for flow cytometric analysis of testicular cells. Representative flow cytometric gating hierarchy used for the quantification of germ cells in cultured testicular tissues (related to Fig. 1g–i).

a Total cell population was identified and gated (R1) based on forward scatter (FSC) and side scatter (SSC) characteristics to exclude debris and minimize background noise.

b Doublet discrimination and single-cell selection. The R1 gated population was further analyzed using propidium iodide (PI) pulse processing. Single cells were selected by gating (R2) based on the relationship between PI-Height (PI-H) and PI-Area (PI-A), effectively excluding cell aggregates. The resulting single-cell population from gate R2 was used for the representative analysis of Acr-GFP fluorescence and DNA content shown in Fig. 1g.

Supplementary Fig. 4

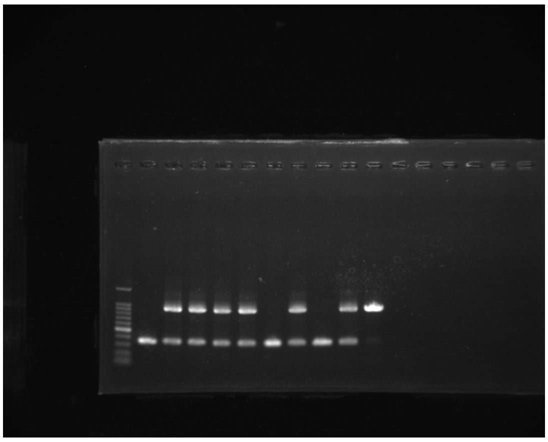

Supplementary Figure 4: Unedited electrophoresis image related to Figure 5c.

Table S1. Results of ROSI experiments

Tissue No.	No. of oocytes injected	No. of 2-cells	No. of 2-cells transferred	No. of implantation	No. of live offspring	Male/ Female
No.1	33	23	23	4	2	0/2
No.2	103	87	58	29	13	5/8

Table S2. Primers used in this study

Target	Purpose	Sequence	Size
Acr-GFP	Genotyping	A:5‘-ACTGAATTGAGTCTACCTGTGGTC-3’<br>B:5‘-GATCATGACTTTGGCCTAATGC-3’<br>C:5‘-CATGGTCCTGCTGGAGTTCGTG-3’	WT(A+B):355bp<br>TG(A+C):924bp
Mael	RT-qPCR	5‘-GAAGCTAAGAGTTGAGAGTCCAGGAT-3’<br>5‘-GATGCTCTCTAGTAAGCGGGTAATTC-3’	160bp
Piwil4	RT-qPCR	5‘-AACCGGTGGTACAGACACAA-3’<br>5‘-CGAGCCGCACTCTGTTACAC-3’	138bp
Tdrd9	RT-qPCR	5‘-TCCAGTGTGACTTTAGAAGAACAGAA-3’<br>5‘-ACATATTTGACATCAGGAACTGTGAC-3’	122bp
LINE1-ORF2	RT-qPCR	5‘-GGAGGGACATTTCATTCTCATCA-3’<br>5‘-GCTGCTCTTGTATTTGGAGCATAGA-3’	87bp
Mvh	RT-qPCR	5‘-TGAAACAGTAGAGACTGAAGCCTTTC-3’<br>5‘-CCACCAAGTACAAAGCTCACATTG-3’	103bp
ORF2	gDNA-qPCR	5‘-CTGGCGAGGATGTGGAGAA-3’<br>5‘-CCTGCAATCCCACCAACAAT-3’	55bp
5S	gDNA-qPCR	5‘-ACGGCCATACCACCCTGAA-3’<br>5‘-GGTCTCCCATCCAAGTACTAACCA-3’	88bp
