# Supplementary material for: Reverse transcriptase inhibitors enable the generation of fertile spermatids from fetal mouse testes in vitro
Source: Commun Biol. 2026 Jan 27;9:329. doi: 10.1038/s42003-026-09613-y (PMC12953628; doi:10.1038/s42003-026-09613-y)
Supplement: Supplementary file 3 — Description of Additional Supplementary Materials [file 42003_2026_9613_MOESM3_ESM.pdf]

Description of Additional Supplementary Files

File name: Supplementary Data 1

Description: Source data underlying the graphs and charts in the main figures
