# Supplementary material for: Reverse transcriptase inhibitors enable the generation of fertile spermatids from fetal mouse testes in vitro
Source: Commun Biol. 2026 Jan 27;9:329. doi: 10.1038/s42003-026-09613-y (PMC12953628; doi:10.1038/s42003-026-09613-y)
Supplement: Supplementary file 5 — Reporting Summary [file 42003_2026_9613_MOESM5_ESM.pdf]

nature portfolio

Corresponding author(s): Takuya Sato

Last updated by author(s): Jan 6, 2026

Reporting Summary

Nature Portfolio wishes to improve the reproducibility of the work that we publish. This form provides structure for consistency and transparency in reporting. For further information on Nature Portfolio policies, see our Editorial Policies and the Editorial Policy Checklist.

Statistics

For all statistical analyses, confirm that the following items are present in the figure legend, table legend, main text, or Methods section.

n/a	Confirmed	
☐	☒	The exact sample size (*n*) for each experimental group/condition, given as a discrete number and unit of measurement
☐	☒	A statement on whether measurements were taken from distinct samples or whether the same sample was measured repeatedly
☐	☒	The statistical test(s) used AND whether they are one- or two-sided<br>*Only common tests should be described solely by name; describe more complex techniques in the Methods section.*
☒	☐	A description of all covariates tested
☒	☐	A description of any assumptions or corrections, such as tests of normality and adjustment for multiple comparisons
☐	☒	A full description of the statistical parameters including central tendency (e.g. means) or other basic estimates (e.g. regression coefficient) AND variation (e.g. standard deviation) or associated estimates of uncertainty (e.g. confidence intervals)
☐	☒	For null hypothesis testing, the test statistic (e.g. *F*, *t*, *r*) with confidence intervals, effect sizes, degrees of freedom and *P* value noted<br>*Give P values as exact values whenever suitable.*
☒	☐	For Bayesian analysis, information on the choice of priors and Markov chain Monte Carlo settings
☒	☐	For hierarchical and complex designs, identification of the appropriate level for tests and full reporting of outcomes
☒	☐	Estimates of effect sizes (e.g. Cohen's *d*, Pearson's *r*), indicating how they were calculated

Our web collection on statistics for biologists contains articles on many of the points above.

Software and code

Policy information about availability of computer code

Data collection	M205 (Leica)<br>BZ-X800 (Keyence)<br>AX (Nikon)<br>StepOne Plus
Data analysis	ImageJ (version 1.54)<br>GraphPad Prism v9<br>StepOne Software v2.3

For manuscripts utilizing custom algorithms or software that are central to the research but not yet described in published literature, software must be made available to editors and reviewers. We strongly encourage code deposition in a community repository (e.g. GitHub). See the Nature Portfolio guidelines for submitting code & software for further information.

Data

Policy information about availability of data

All manuscripts must include a data availability statement. This statement should provide the following information, where applicable:

- Accession codes, unique identifiers, or web links for publicly available datasets
- A description of any restrictions on data availability
- For clinical datasets or third party data, please ensure that the statement adheres to our policy

Provide your data availability statement here.

Research involving human participants, their data, or biological material

Policy information about studies with human participants or human data. See also policy information about sex, gender (identity/presentation), and sexual orientation and race, ethnicity and racism.

Reporting on sex and gender	*Use the terms sex (biological attribute) and gender (shaped by social and cultural circumstances) carefully in order to avoid confusing both terms. Indicate if findings apply to only one sex or gender; describe whether sex and gender were considered in study design; whether sex and/or gender was determined based on self-reporting or assigned and methods used. Provide in the source data disaggregated sex and gender data, where this information has been collected, and if consent has been obtained for sharing of individual-level data; provide overall numbers in this Reporting Summary. Please state if this information has not been collected. Report sex- and gender-based analyses where performed, justify reasons for lack of sex- and gender-based analysis.*
Reporting on race, ethnicity, or other socially relevant groupings	*Please specify the socially constructed or socially relevant categorization variable(s) used in your manuscript and explain why they were used. Please note that such variables should not be used as proxies for other socially constructed/relevant variables (for example, race or ethnicity should not be used as a proxy for socioeconomic status). Provide clear definitions of the relevant terms used, how they were provided (by the participants/respondents, the researchers, or third parties), and the method(s) used to classify people into the different categories (e.g. self-report, census or administrative data, social media data, etc.) Please provide details about how you controlled for confounding variables in your analyses.*
Population characteristics	*Describe the covariate-relevant population characteristics of the human research participants (e.g. age, genotypic information, past and current diagnosis and treatment categories). If you filled out the behavioural & social sciences study design questions and have nothing to add here, write "See above."*
Recruitment	*Describe how participants were recruited. Outline any potential self-selection bias or other biases that may be present and how these are likely to impact results.*
Ethics oversight	*Identify the organization(s) that approved the study protocol.*

Note that full information on the approval of the study protocol must also be provided in the manuscript.

Field-specific reporting

Please select the one below that is the best fit for your research. If you are not sure, read the appropriate sections before making your selection.

☒ Life sciences ☐ Behavioural & social sciences ☐ Ecological, evolutionary & environmental sciences

For a reference copy of the document with all sections, see nature.com/documents/nr-reporting-summary-flat.pdf

Life sciences study design

All studies must disclose on these points even when the disclosure is negative.

Sample size	No statistical methods were used to predetermine sample size.
Data exclusions	No data was excluded from the analyses.
Replication	Experiments were conducted at least three independent times, with the exception of the data in Figures 1C, 1E, 3F, and for specific conditions within Figure 1B (d4T 50, AZT 10, EFV 0.1, ADV 0.1–1, and ddC 1–100). These specific datasets are based on one or two independent replicates. In the graphs, each dot represents data from a single analyzed testis or testicular fragment.
Randomization	Animals were distributed at random over experimental groups.
Blinding	Blinding was not possible.

Reporting for specific materials, systems and methods

We require information from authors about some types of materials, experimental systems and methods used in many studies. Here, indicate whether each material, system or method listed is relevant to your study. If you are not sure if a list item applies to your research, read the appropriate section before selecting a response.

Materials & experimental systems

n/a	Involved in the study
☐	☒ Antibodies
☒	☐ Eukaryotic cell lines
☒	☐ Palaeontology and archaeology
☐	☒ Animals and other organisms
☒	☐ Clinical data
☒	☐ Dual use research of concern
☒	☐ Plants

Methods

n/a	Involved in the study
☒	☐ ChIP-seq
☐	☒ Flow cytometry
☒	☐ MRI-based neuroimaging

Antibodies

Antibodies used	Chicken anti-GFP (abcam, cat no. ab13970, lot no. GR3361051-13)<br>Rabbit anti-LINE1-ORF1p (abcam no. ab216324, lot no. 1011448-1)<br>Alexa Fluor 488 Goat anti-Chicken (Life Technologies, cat no. A11039, lot no. 2566343)<br>Alexa Fluor 488-conjugated polyclonal GFP antibody (Life Technologies, cat no. A-21311, lot no. 2207528)
Validation	Chicken anti-GFP was validated by the manufacturer (https://www.abcam.co.jp/products/primary-antibodies/gfp-antibody-ab13970.html).<br>Rabbit anti-LINE1-ORF1p was validated by the manufacturer (https://www.abcam.co.jp/products/primary-antibodies/line-1-orf1p-antibody-epr21844-108-ab216324.html)<br>Alexa Fluor 488 Goat anti-Chicken was validated by the manufacturer (https://www.thermofisher.com/antibody/product/Goat-anti-Chicken-IgY-H-L-Secondary-Antibody-Polyclonal/A-11039).<br>Alexa Fluor 488-conjugated polyclonal GFP antibody was validated by the manufacturer (https://www.thermofisher.com/antibody/product/GFP-Antibody-Polyclonal/A-21311).

Animals and other research organisms

Policy information about studies involving animals; ARRIVE guidelines recommended for reporting animal research, and Sex and Gender in Research

Laboratory animals	Acr-GFP Tg mice (strain: ICR (CLEA Japan Inc.), C57BL/6 (CLEA Japan Inc.) and their mixture) were supplied by RIKEN BRC. In the postnatal mouse testis culture experiment, it was used at 1.5-5.5 dpp. In the fetal testis culture experiment, homozygous male Acr-GFP and ICR females were mated and used at E12.5 or E14.5.
Wild animals	Our study did not involve wild animals.
Reporting on sex	*Indicate if findings apply to only one sex; describe whether sex was considered in study design, methods used for assigning sex. Provide data disaggregated for sex where this information has been collected in the source data as appropriate; provide overall numbers in this Reporting Summary. Please state if this information has not been collected. Report sex-based analyses where performed, justify reasons for lack of sex-based analysis.*
Field-collected samples	Our study did not involve Field-collected samples.
Ethics oversight	This study was performed under in accordance with protocols approved by the Institutional Committee of Laboratory Animal Experimentation (Animal Research Center of Yokohama City University; protocol no. F-A-23-023).

Note that full information on the approval of the study protocol must also be provided in the manuscript.

Plants

Seed stocks

Report on the source of all seed stocks or other plant material used. If applicable, state the seed stock centre and catalogue number. If plant specimens were collected from the field, describe the collection location, date and sampling procedures.

Novel plant genotypes

Describe the methods by which all novel plant genotypes were produced. This includes those generated by transgenic approaches, gene editing, chemical/radiation-based mutagenesis and hybridization. For transgenic lines, describe the transformation method, the number of independent lines analyzed and the generation upon which experiments were performed. For gene-edited lines, describe the editor used, the endogenous sequence targeted for editing, the targeting guide RNA sequence (if applicable) and how the editor was applied.

Authentication

Describe any authentication procedures for each seed stock used or novel genotype generated. Describe any experiments used to assess the effect of a mutation and, where applicable, how potential secondary effects (e.g. second site T-DNA insertions, mosiacism, off-target gene editing) were examined.

Flow Cytometry

Plots

Confirm that:

☒ The axis labels state the marker and fluorochrome used (e.g. CD4-FITC).

☒ The axis scales are clearly visible. Include numbers along axes only for bottom left plot of group (a 'group' is an analysis of identical markers).

☒ All plots are contour plots with outliers or pseudocolor plots.

☒ A numerical value for number of cells or percentage (with statistics) is provided.

Methodology

Sample preparation

Sample preparation was described in the manuscript.

Instrument

Guava easyCyte flow cytometer

Software

InCyte 3.3

Cell population abundance

Cell sorting was not performed.

Gating strategy

FSC-H and SSC-H scatter plot were used to separate cell events from debris. PI-H and PI-A were used to separate singlets and doublets.

☒ Tick this box to confirm that a figure exemplifying the gating strategy is provided in the Supplementary Information.
